# Human adipose derived stem cells regress fibrosis in a chronic renal fibrotic model induced by adenine

**DOI:** 10.1371/journal.pone.0187907

**Published:** 2017-12-27

**Authors:** Juan José Rivera-Valdés, Jesus García-Bañuelos, Adriana Salazar-Montes, Leonel García-Benavides, Alfredo Rosales-Dominguez, Juan Armendáriz-Borunda, Ana Sandoval-Rodríguez

**Affiliations:** 1 Institute for Molecular Biology in Medicine and Gene Therapy, Department of Molecular Biology and Genomics, Health Sciences University Center, University of Guadalajara, Guadalajara, Jalisco, Mexico; 2 Department of Biomedical Sciences, Tonala University Center, University of Guadalajara, Tonala, Jalisco, Mexico; 3 Chronic-Degenerative Diseases Institute, Health Sciences University Center, University of Guadalajara, Guadalajara, Jalisco, Mexico; 4 Tecnologico de Monterrey, Guadalajara, Jalisco, Mexico; University Medical Center Utrecht, NETHERLANDS

## Abstract

**Background and aims:**

ADSCs transplantation had been shown in some experimental models of kidney damage that it improves kidney function and reduces fibrosis. In this study we evaluated the effect of human adipose tissue-derived stem cell (hADSC) therapy in a chronic kidney damage experimental model.

**Methods:**

A chronic kidney injury was induced by daily orogastric administration of adenine (100mg/kg) to male Wistar rats for 28 days. hADSCs were isolated, expanded and characterized before transplantation. hADSC administration was performed in a tail vein at a dose of 2 x10^6^ cells/animal. Animals were sacrificed at 7 days post-treatment. The percentage of fibrotic tissue, serum and urine levels of urea, creatinine, total protein and renal mRNA of COL1A1, TGFB1, CTGF, ACTA2, IL6, IL10, TNF were analyzed.

**Results:**

hADSCs treatment significantly reduces kidney fibrosis, improves urea and creatinine serum and urine levels, and diminishes COL1A1, TGFB1, CTGF, ACTA2 mRNA kidney levels.

**Conclusions:**

These results showed that cell therapy using hADSCs improves renal function and reduces fibrosis.

## Introduction

Renal fibrosis (RF) occurs as a natural wound-healing process in response to several injuries [1]. The common final outcome of almost all progressive chronic kidney diseases (CKD) caused by diabetes or hypertension is tubulointerstitial fibrosis [1]. The most remarkable events in tubulointerstitial fibrosis include: first, inflammatory cell infiltration; second, activation and expansion of fibroblasts; third, the production and deposition of a wide amount of components of extracellular matrix (ECM), especially fibronectin and collagen type I and type III, and finally tubular atrophy and microvascular malfunction. Altogether they lead to the destruction of renal parenchyma and consequently a loss of function in the organ [2]. The experimental model of chronic intoxication with adenine resembles metabolic abnormalities observed in CDK in humans [3]. Adenine is a nitrogen heterocycle that is efficiently salvaged by adenine phosphoribosyltransferase [4]. When adenine is present in excess, it is oxidized into 2,8-dihydroxyadenine; both are excreted in urine [5]. Nevertheless, the very low solubility of 2,8-dihydroxyadenine can lead to its precipitation in the tubules [6], forming crystals that prevent proper functioning of the kidney, leading to the characteristic events of CKD. At this time there is no efficient treatment against renal fibrosis or renal failure. Kidney transplantation in some cases could be an option but its high cost, shortage of donors and the possibility of organ rejection constrain its widespread application, especially in underdeveloped countries. Therefore, the search for new therapies to hinder CKD at early stages represents a mandatory issue.

Adipose tissue-derived stem cells (ADSC) are a type of mesenchymal stem cells, which in culture conditions exhibit strong adherence to plastic and a fusiform morphology. Currently, ADSC have been used as a therapeutic strategy for injured organ regeneration in animal models and in a few clinical trials. Beneficial effects of ADSCs are attributed to their extensive secretome that includes interleukins such as IL-6, IL-7, IL-8, IL-11, IL-10, growth factors *i*.*e*. VEGF, HGF, GM-CSF, bFGF, BDNF, IGF-1 and other proteins that are suitable inducers of tissue regeneration [7–10]. Some studies have employed ADSC as a therapeutic strategy in kidneys by using ischemia, folic acid nephrotoxicity or nephrectomy to induce damage [11–13]. Described beneficial effects include reduced serum urea, kidney fibrosis and chronic inflammation; demonstrated by reduced interstitial collagen deposition, tissue chemokine and cytokine expression [11]. In the Furuichi et al. study, repeated administration of ADSCs reduced acute tubular necrosis and interstitial macrophage infiltration in the injured kidney, and reduced cytokine and chemokine expression [14]. Other reported effects include reduction of plasma creatinine levels, lower expression of damage markers ED-1 and α-SMA; also, treated rats improved renal function [15]. Then, the aim of this study was to evaluate the potential antifibrotic effect of human hADSC in an experimental model of chronic kidney damage induced by adenine.

## Materials and methods

### hADSCs isolation and culture

Human adipose tissue was obtained from the abdominal fat of a female subjected to cosmetic liposuction who previously signed an informed consent regarding donation of her fat for research. The protocol was approved by the Research and Ethical Committee of CUCS, University of Guadalajara (approval number C.I. 005–2017) which approved the fat obtainment procedure. Tissue was digested by a 0.075% collagenase type II solution (Invitrogen, Grand Island, NY) gently shaken for 1 hour at 37°C. Digestion product was filtered using a 100 um nylon mesh and centrifuged at 1200 g for 8 min. Pellet was washed with PBS and erythrocytes were lysed. Cells were collected and plated on plastic dishes in DMEM (Invitrogen, Grand Island, NY) supplemented with 10% fetal bovine serum (Invitrogen, Grand Island, NY) and 1% antibiotic (Invitrogen, Grand Island, NY). The medium was changed after 48 h. Cells were harvested and seeded until passage 3 to achieve greater expansion. Cell characterization and transplantation were made in this unique batch of cells obtained from one single fat donor in a sole isolation.

### hADSCs characterization and *in vitro* differentiation

Surface markers for hADSCs were evaluated using a mini Guava EasyCyte flow cytometer. 5x10^4^ cells were incubated with fluorescein isothiocyanate (FITC) or phycoerythrin (PE)-conjugated antibodies, anti-CD105, anti-CD34, anti-STROI, anti-CD73, anti-CD45, anti-HLA-ABC, anti-HLA-DR (Invitrogen, Frederick, MD) for 30 min at 4°C in PBS and washed afterwards. Cell autofluorescence in channel F1 or F2 was subtracted to obtain a neat signal of each marker. 5x10^4^ cells were plated for differentiation tests. Adipogenic differentiation was performed in StemPro adipogenesis-conditioned medium (Invitrogen, Grand Island, NY) for one week, replacing medium every 3 days. Lipid droplets were stained by using red oil staining for validating differentiation. StemPro osteogenesis-conditioned medium (Invitrogen, Grand Island, NY) was used for osteogenic differentiation for 11 days, replacing media every 48 hours. Extracellular calcium deposits were identified by Von Kossa staining. hADSCs cultured in DMEN were also stained as controls.

### Animal model of chronic kidney disease and hADSCs transplantation

Twelve male Wistar rats (n = 6/group, ∼250 g) were intoxicated orogastrically with adenine (100 mg/kg) for 4 weeks, a modification of the Yokozawa *et al*. animal model for chronic renal failure [3]. Six non-manipulated rats served as the healthy control (n = 6). The treated group (n = 6) was administrated 2x10^6^ hADSCs via a tail vein 24 h after final adenine administration. On day 6 posttreatment, each rat was weighed and housed individually for 24 h for urine collection. All animals were sacrificed 7 days posttreatment; kidneys and blood were collected. The right kidney was used for histological assays while the left kidney was used for molecular analysis. Animals were obtained from the Animal Facility at the University Center for Health Sciences of the University of Guadalajara and housed in a maximum of 4 animals/cage. Rats received care according to Official Mexican Norm NOM-062-ZOO-1999 and guidelines of the Animal Facility at the University Center for Health Sciences of the University of Guadalajara, using 12h light/dark cycles. Rats were fed *ad libitum* and had free access to water. Health of the animals was monitored daily. When animals were identified in pain during the adenine intoxication, had ear infections, showed slow or not movement, had brittle hair and eye dehydration, they were euthanized by applying an overdose (100mg/kg) of intraperitoneally anesthesia (Zoletil® 100; Tiletamine 50mg/ml and Zolazepam 50mg/ml). No animals died prior to the experiment endpoint. The protocol was approved by the Research and Ethical Committees of CUCS, University of Guadalajara (approval number C.I. 005–2017), which reviewed the animal mortality aspects of the protocol.

### Analysis of kidney tissue specimens

The right kidney was removed after sacrifice and fixed by immersion in a 10% paraformaldehyde solution, dehydrated in ethylic alcohol and embedded in paraffin. Sections 5μm thick were stained with Masson´s trichrome, Hematoxylin & Eosin and Sirius Red. Percentage of fibrotic tissue was determined in 30 microphotographs using a computer-assisted image analyzer (Image-ProPlus 6.0, Media Cybernetics, Inc., Bethesda, MD). In addition, the Banff Classification of Kidney Allograft Pathology [16] was used by a pathologist blinded to the study to evaluate the degree of interstitial fibrosis, tubular atrophy, interstitial inflammation and mesangial matrix increase. Kidney tissue RNA isolation was performed according to the Chomczynski and Sacchi modified method [17]. Briefly, kidney tissue was homogenized in the presence of a Trizol reagent (Invitrogen, Carlsbad, CA). Chloroform was added and the aqueous phase was isolated. RNA was precipitated with isopropanol. RNA quantity and quality were determined with NanoDrop equipment (Thermo Scientific, USA). 2 μg of total RNA were used for retrotranscription with 240 ng Oligo dT, 0.5 mM dNTPs mix, 10mM DTT, 2 U of RNAse inhibitor and 200 U M-MLV (Invitrogen, Carlsbad, CA). Incubation was performed for 10 min at 25°C, 50 min at 37°C, 15 min at 70°C and 5 min in ice. The samples were stored at -70°C until used. Using a LightCycler 96 instrument (Roche Diagnostics, Indianapolis, IN), qPCR was performed as follows: 1 cycle at 50°C for 2 min, 1 cycle at 95°C for 5 min, and 30–40 cycles at 95°C for 30 s and 60°C for 40 s. The total reaction volume was 10 μL containing 2 μL of cDNA, 1X Universal PCR Master Mix (Roche, Branchburg, NJ) and 1X TaqMan primers/probe (Applied Biosystems, Foster City, CA). mRNA Levels of COL1A1, TGFB1, CTGF, ACTA2, IL6, IL10, TNF were normalized using 18s RNA as a housekeeping gene. Data analysis was performed using the 2-Δct method [18].

### Evaluation of renal function

Blood was collected at the time the animals were sacrificed and serum was obtained. Serum values of BUN, creatinine as well as 24h-urine levels of creatinine and total protein were evaluated using a Vitros 250 automatic analyzer (Johnson and Johnson, New Jersey, NY).

### Statistic analysis

Values are expressed as mean ± SD. Groups were compared with one way ANOVA followed by Bonferroni test. Values of p <0.05 were considered statistically significant. An analysis was run with GraphPad Prism version 6.

## Results

### Animal model for chronic renal failure

Oral administration of adenine is metabolized to 2,8-dihydroxyadenine, which precipitates and forms crystals in the microvilli and apical epithelial region of the proximal tubule causing degenerative changes in renal tubules and the interstitium [19].

**Fig 1:**
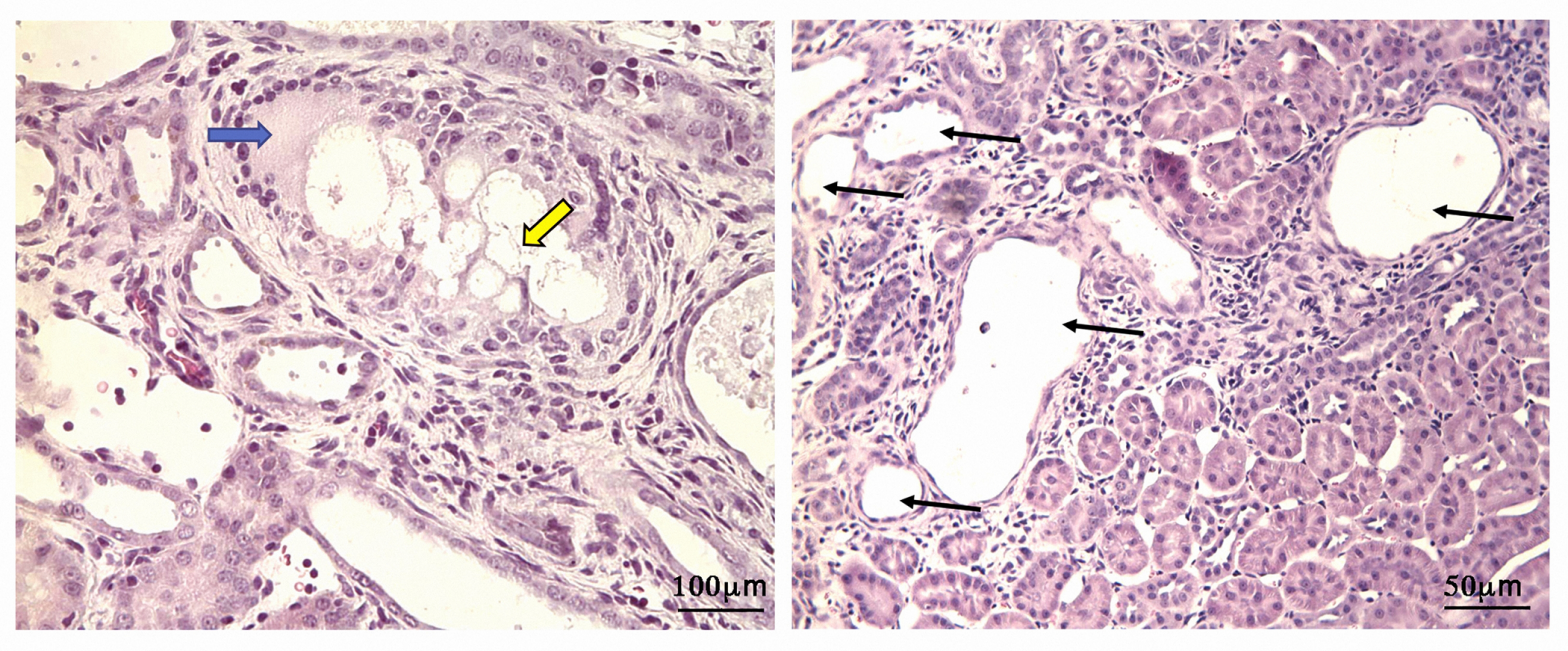
Renal damage induced by chronic administration of adenine. A) Rat kidney tissue showing aggregates of adenine crystals (yellow arrow) surrounded by epithelioid histiocytes, with accompanying multinucleated giant cells (blue arrow). hematoxylin-eosin, original magnifications X400. B) renal cortex segment showing dilated renal tubules stained with hematoxylin and eosin (arrows) in adenine-treated rats (X200 magnification).

Long-term feeding of adenine to rats produced metabolic abnormalities resembling chronic renal failure in humans. The pathological findings in the kidneys of our experimental rats revealed lesions of proximal tubules, of some distal tubules and of glomeruli (Fig 1).

### Characterization of hADSC

**Fig 2:**
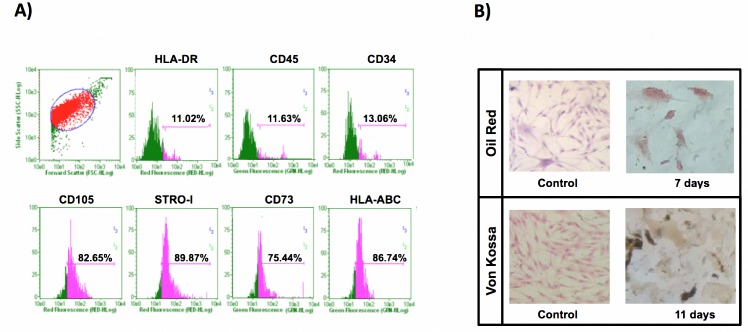
Characterization of hADSC. A) Phenotype characterization using flow cytometry analysis was performed at third pass. Cell population was positive for the CD105 marker in 82.65%, STRO-I 89.87%, CD73 with 75.44% and HLA-ABC in 86.74%; and negative for HLA-DR with a 11.02% presence, CD45 with 11.63%, and CD-34 marker showed 13.06% of positivity. B) Functional characterization was validated by using commercial culture media. Adipogenic differentiation was verified by using red oil staining; lipid vacuoles were stained red (40X). Osteogenic differentiated cells were detected with Von Kossa staining, showing extracellular calcium deposits as brown spots. Control hADSCs in standard growth culture media displayed their characteristic fibroblast morphology (20X).

Cultured hADSCs isolated by the enzymatic digestion method showed plastic adherence and typical fibroblast-like morphology. When cells were at the third passage, flow cytometry was performed and cell markers CD73, CD-105, STRO-I and HLA-ABC were positive while CD34, CD45 and HLA-DR were negative (Fig 2A). Cells were cultured in commercial differentiation-inducing media and showed an ability to differentiate osteocytes and adipocytes. Fig 2B validates adipocyte differentiation through red oil staining and osteocyte differentiation using Von Kossa staining. Red-stained intracellular lipid vacuoles were observed under microscope only in the adipogenic differentiated culture; lipid vacuoles were not observed in control cells in DMEM medium. In addition, Fig 2B showed brown extracellular calcium deposits characteristic of the osteogenic lineage in Von Kossa staining. Control cells did not show calcium deposits.

### hADSC transplantation decreased renal fibrosis

Histopathological analysis was performed by a pathologist blind to the study based on the Banff classification of renal allograft pathology. The morphologically fibrotic group showed kidney tissue with characteristics of renal damage such as: tubular necrosis, tissue impairment, inflammatory cell infiltration and ECM deposition. On the other hand, treated animals showed a decrease in ECM staining and improvement in tissue architecture and tubular structure. The pathologist’s analysis indicated that the fibrotic group was positioned in scores 2–3 for interstitial infiltrate parameter, whereas those of the treated group scored 1–2, showing a slight decrease in inflammatory infiltrate without reaching significant differences. As for the interstitial fibrosis parameter, the fibrotic control group scored 2–3 with very high percentages of fibrosis in the cortical area (48.2±12.6%). A significant decrease was observed in the hADCSs group with samples positioned in scores 1–2 with very low percentages (25.6%±4.2%) of fibrosis (P<0.01). Tubular atrophy showed a moderate improvement when hADSC was administered, from scores of 2–3 in the fibrotic group to scores of 1–2 in the ADSCs group. Finally, no differences were found between the two groups in terms of mesangial expansion. Table 1.

**Table 1:** Banff classification for renal damage.

BANFF CLASSIFICATION				
	Histological Indicators of Renal Damage			
Group	Interstitial Fibrosis	Tubular Atrophy	Interstitial Inflammation	Mesangial Matrix Increase
**Healthy**	**0** (2.05% ± 1.7)	**0** (0.8% ± 0.9)	**0** (5.6% ± 2.1)	**0** (1% ± 0.5)
**Fibrotic**	**2.2** (48.2% ± 12.6)	**2.2** (39.8% ± 8.5)	**2** (42.8% ± 24.3)	**2.8** (69.2% ± 18.1)
**hADSC**	**1.6** (25.6% ± 4.2)	**1.6** (25.67% ± 17.2)	**1.6** (26.67% ± 19.2)	**3** (62.67% ± 7.6)

**Fig 3:**
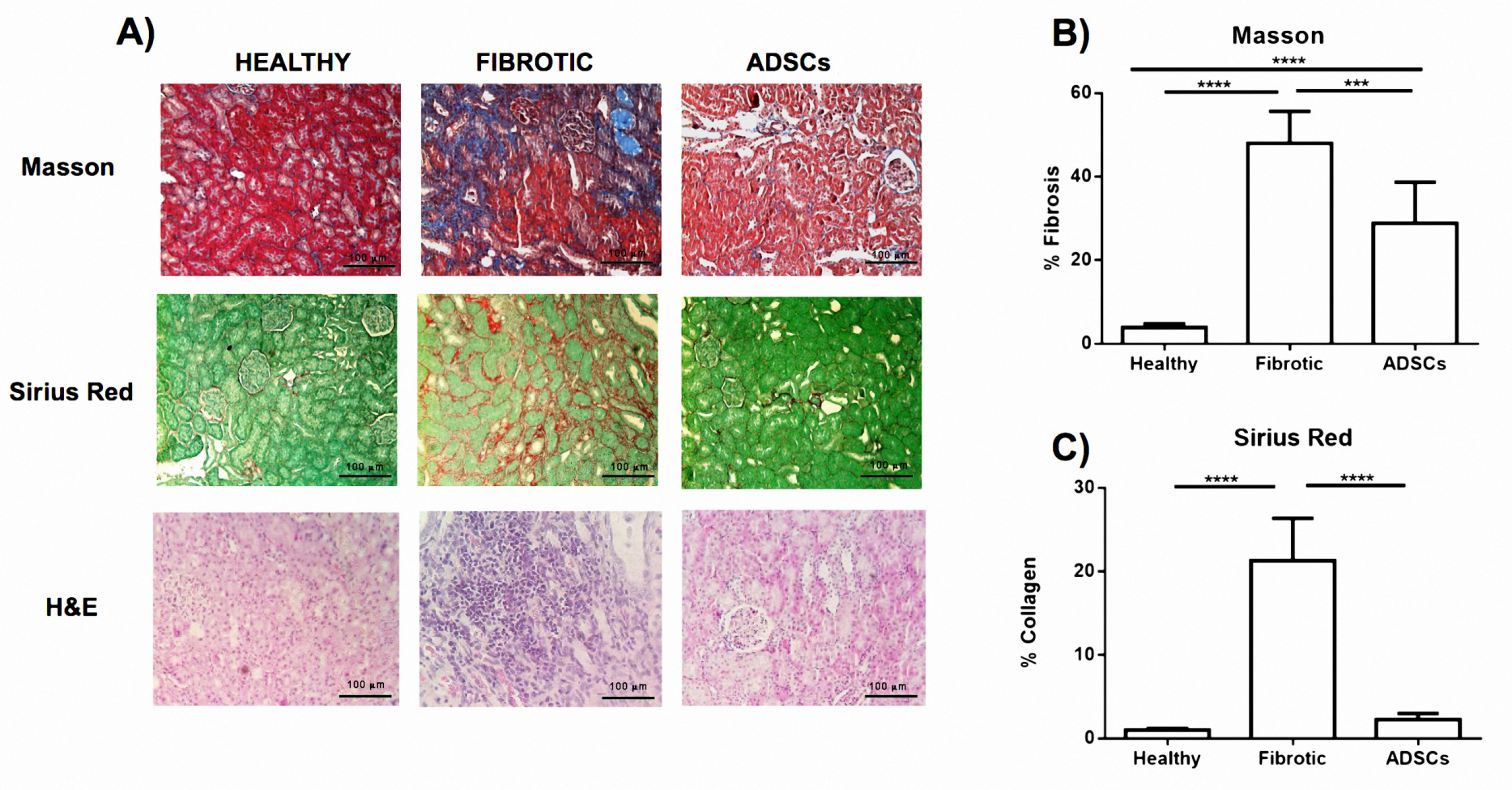
hADSC transplantation decreased renal fibrosis. A) Representative pictures of renal sections stained with Masson's trichrome (40X), Sirius Red (40X) and Hematoxylin & Eosin (20X). B) Morphometric analysis of microphotographs of Masson and Sirius red stained tissue to evaluate fibrosis and collagen deposition. Data represent a mean ± SD. (**p<0.01, ***p<0.001, ****p<0.0001).

3–5 μm-thick kidney tissue was subjected to Masson’s trichrome and Sirius red staining to quantify the percentage of fibrosis, allowing us to distinguish the extracellular matrix and collagen I respectively of the nucleus and cytoplasm. Image analysis calculated the area of fibrotic tissue and the area of renal parenchyma based on pixel quantification. Masson staining analysis showed 3.8±0.9% in the healthy control, 48.0±7.7% in fibrotic animals and 28.8±9.9% (p <0.001) in the hADSC group, indicating a 40% reduction in ECM installation. Regarding the collagen content, Sirius red staining indicated a percentage of 1.1±0.2% in the healthy group, 21.3± 5.1% in the fibrotic group and 2.3±0.7% in the treated group (p <0.001). Reduction of collagen deposition was 89% (Fig 3).

### hADSC treatment diminished fibrogenic gene profile

The expression levels of profibrogenic genes such as COL1A1, TGFB1, CTGF, ACTA2 were evaluated. Likewise, the expression levels of proinflammatory genes TNF, IL6; and IL-10 as an anti-inflammatory molecule were determined. COL1A1 and ACTA2 mRNA decreased significantly (p <0.05) in the group treated with a single dose of hADSC when compared to the fibrotic control group. In the case of TGFB1, a significant difference (p <0.001) was observed compared to control. However, expression levels for the CTGF gene were not statistically different (NS) between the study groups.

**Fig 4:**
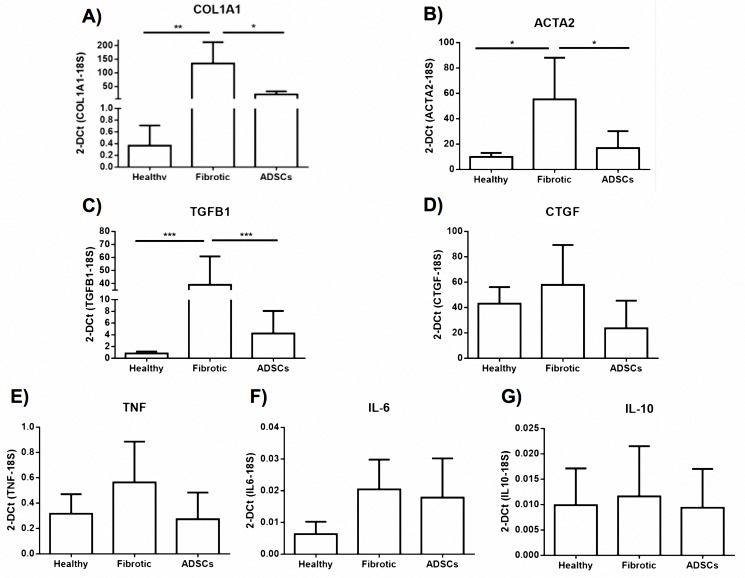
Gene expression of fibrogenic and inflammatory molecules. Administration of a single dose of hADSCs downregulated the mRNA expression of profibrogenic and proinflammatory molecules. COL1A1, TGFB1 and ACTA2 reached statistical significance. The data represented a mean ± SD. (*p<0.05, **p<0.01, ***p<0.001).

Regarding the levels of expression at the mRNA level of the proinflammatory genes, TNF and IL-6, despite being elevated in the fibrotic group with respect to the control group, no significant differences were observed, whereas in the treated group a tendency towards the decrease and normalization of the expression of these proinflammatory genes can be seen since it shows a greater similarity to levels reported in the healthy control group. Finally, expression of IL-10, the anti-inflammatory cytokine, does not differ among the three study groups, probably because the fibrotic model occurs during a 28-day, chronic non-acute adenine administration (Fig 4).

The fibrosis model with chronic administration of adenine managed to overexpress profibrogenic genes; these differences were statistically significant in comparison with the healthy control group. In other words, results seen here show significances (p <0.01) for the expression of COL1A1, (p <0.05) for ACTA2, and (p <0.001) TGFB1. In the case of CTGF, in spite of not presenting a significant difference between the fibrotic group and the control group, a strong tendency towards the increase of this gene was observed in the group submitted to the experimental model.

### Cell therapy improved renal function

Chronic adenine administration was able to induce renal damage at 28 days, and increased serologic levels of urea and creatinine compared to the control group (p<0.01), confirming that this model successfully resembles the biochemical changes experienced by patients with renal failure [20].

**Fig 5:**
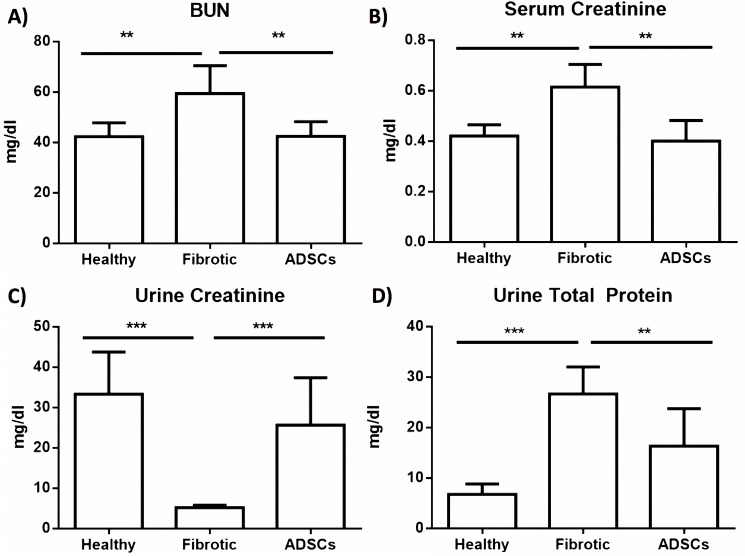
ADSCs treatment improved biochemical markers of renal function. A) and B) Serum levels of BUN and creatinine showed a significant reduction after cell therapy. C) and D) urine levels of creatinine and total protein increased after cell transplantation. The data represent a mean ± SD. (*p<0.05, **p<0.01, ***p<0.001).

hADSCs treatment significantly improved urea and creatinine serum levels and total urine protein content. BUN data showed a significant reduction in the ADSCs group with 42.9±5.9 while the fibrotic group was 59.4±11.1 mg/dl (p<0.01). Healthy animals were at 42.3±5.5mg/dl (Fig 5A). There was a significant decrease of Creatinine in blood levels and an increase in its urine content. Serum creatinine in healthy animals was 0.42±0.04, in the non-treated group it was 0.61±0.08 while in treated animals it was 0.4±0.08 mg/dl; therefore this reduction achieved statistical significance (p<0.01) (Fig 5B). At the same time, as seen in Fig 5C, urine levels of creatinine increased, indicating an improvement of renal function. The ADSCs group showed 25.6±11.8 mg/dl levels similar to those found in healthy animals, at 33.3±10.4 mg/dl. Compared to 5.2±0.6 mg/dl in control fibrotic group, levels in treated animals reached statistical significance (p<0.001). Also, Fig 5D shows an improved total urine protein determination in the treated group, compared to the fibrotic control. Values reached 6.8±2.1 in healthy animals, 26.7±5.3 in fibrotics while16.3±7.5 mg/dl in the treated group, showing statistical significance (p<0.01).

## Discussion

In this study we show that systemic application of hADSCs results in the amelioration of kidney fibrosis, reducing the amount of fibrotic tissue, improving renal function and decreasing gene expression of profibrogenic molecules. The antifibrogenic effect of MSCs has been reported in different organs such as lung [21], liver [22] and kidney [23]. Several studies center on the effects of stem cell therapy in acute kidney damage; most of them use animal autologous transplantation [11,14,24,25], few of them use hADSC [26–29], and even fewer test them on CKD models [15]. In these studies, cell origin, administration routes and animal models vary, but coincide in a helpful effect of cell-based therapies for kidney fibrosis. Furuichi et al. explored the effect of mADSC in a mouse model of acute renal impairment due to ischemia, achieving reduction of tubular necrosis and interstitial infiltration of macrophages, with no antifibrogenic effect 7 days posttreatment [14]. In this regard, in our study a single administration of hADSC (2x10^6^ cells) achieved a 40% decrease in fibrosis compared to the control group. Also, Collagen staining corroborated this reduction, indicating 89% less collagen deposition in treated animals and indicating extensive improvement in renal tissue due to treatment. These data are similar to Burgos et al. who evaluated autologous transplantation of ADSCs in FVB mice after inducing acute renal damage with folic acid. Treatment with mADSCs was effective in preventing collagen deposition and reducing tissue fibrosis by about 60% [11]. In our study, a histological decrease in fibrosis correlates with the gene expression analysis where significant downregulation of profibrogenic molecules such as COL1A1, ACTA2 y TGFB1 was observed. CTGF also decreased despite not reaching a significant difference. We believe that hADSCs have clear advantages as therapy in CKD, reducing α-SMA mRNA, and decreasing COL1A1, TGFB1 and CTGF mRNA levels. hADSC administration was also able to restore levels of the biological markers of renal damage, creatinine, BUN and total protein within a week of being injected. These results indicate a clear beneficial effect of hADSC transplantation in kidney damage.

The aim of this study was to evaluate a possible beneficial effect of the transplantation of ADSCs. We did not go any deeper in mechanisms behind the anti-fibrotic effect we observed. However, different studies have reported renal engraftment [30–32] in several models and conditions. Other studies have shown that ADSCs secretome includes multiple growth factors, such as VEGF, HGF, GM-CSF, bFGF, BDNF and IGF-1 and interleukins, such as IL-6, IL-7, IL-8, IL-11, IL-10, etc. that contribute to restore microenvironment in the transplanted tissue [33,34].

Particularly, in renal studies MSC have shown renoprotection via local paracrine action. Bi et al. described that intraperitoneal administration of conditioned media from cultured stromal cells induced migration and proliferation of kidney-derived epithelial cells and significantly diminished cisplatin-induced proximal tubule cell death in vitro, increased survival, and limited renal injury [35]. Tögel et al, established that VEGF is a critical factor mediating renal recovery. VEGF knockdown by small-interfering RNA reduced significantly the effectiveness of administered MSCs and decreased survival. Also, 3 months postadministration, MSCs were not engrafted in any tissues except in the bone marrow in 50% of animals given the highest allogeneic cell dose [36]. Tomasoni et al suggested that horizontal transfer of the mRNA for IGF-1R to tubular cells through exosomes or microvesicles derived from MSCs, potentiates tubular cell sensitivity to locally produced IGF-1 providing a new mechanism underlying the powerful renoprotection of BM-MSC observed in vivo [37]. Taking this into account, it is fair to assume that ADSCs paracrine effects might have provided the therapeutic effects observed in this study. Mechanisms proposed for the ADSC antifibrotic effect include several cytokines such as IL-10 and HGF, with paracrine influence on activated fibroblast [38,39]. This assumption correlates with our results for kidney α-SMA mRNA levels that diminish, probably due to a decrease in fibroblast activation. Furthermore, we did not make any attempt to track ADSCs in this study; but some authors have reported that MSC administered systemically migrate to renal tissue in remnant kidney model and acute renal failure, through CD44-Hyaluronic acid [30,31].

Our results show that kidney inflammation after hADSCs transplantation was not modified. Histologically, the Banff classification for renal allograft pathology was used by a pathologist blind to the study who did not find statistical differences between groups. On the other hand, mRNA of the proinflamatory molecule TNF tends to reduce its level in treated animals when compared to the control group; however, no statistical significance was achieved. Treatment time should probably be longer to be able to observe modifications of this parameter.

ADSCs secretome is the principal mechanism suggested for mediating the antifibrogenic effect; VEGF, HGF, FGF-2, among other growth factors, could promote angiogenesis and renal regeneration processes [40,41]; and matrix metalloproteinases (MMPs) [8] that are traditionally conceived as antifibrotic agents in the development and progression of chronic kidney diseases (CKD). This secretome could lead to a microenvironment improvement of the kidney tissue that could be assisting renal regeneration [42].

In conclusion, we consider that our data support the use of ADSCs for renal pathologies associated with fibrosis, due to its efficient antifibrotic effect in this organ when administered in caudal in animals with chronic renal damage. Future work should be done to show with certainty the molecules responsible for this therapeutic effect *in vivo*.

## Supporting information

S1 File Commercial Taqman probe/primers for real time PCR (life technologies). GAPDH: glyceraldehyde-3-phosphate dehydrogenase; COL1A1: collagen, type I, alpha 1 chain; TGFB1: transforming growth factor-β1; CTGF: connective tissue growth factor; MMP2: matrix metallopeptidase 2; ACTA2: smooth muscle alpha-actin 1; PAI1A: plasminogen activator inhibitor type.
(DOCX)

S2 File Descriptive statistics of biochemical tests, morphometric analysis and gene expression profile. The data represent a mean, median, SD and SE for for each parameter of each group.
(XLS)

S3 File Body weight of animals during the experimental model. The body weight of each group was determinated since the beginning of the model (week 0) to the end of the study (week 5). The data are presented as mean and SD.
(XLS)
