# Correction: Human adipose derived stem cells regress fibrosis in a chronic renal fibrotic model induced by adenine

**DOI:** 10.1371/journal.pone.0196045

**Published:** 2018-04-13

**Authors:** Juan José Rivera-Valdés, Jesus García-Bañuelos, Adriana Salazar-Montes, Leonel García-Benavides, Alfredo Dominguez-Rosales, Juan Armendáriz-Borunda, Ana Sandoval-Rodríguez

There is an error in the fifth author’s last name. The correct name is: Alfredo Dominguez-Rosales. The correct citation is: Rivera-Valdés JJ, García-Bañuelos J, Salazar-Montes A, García-Benavides L, Dominguez-Rosales A, Armendáriz-Borunda J, et al. (2017) Human adipose derived stem cells regress fibrosis in a chronic renal fibrotic model induced by adenine. PLoS ONE 12(12): e0187907. https://doi.org/10.1371/journal.pone.0187907
