# Supplementary material for: Human adipose derived stem cells regress fibrosis in a chronic renal fibrotic model induced by adenine
Source: PLoS One. 2017 Dec 27;12(12):e0187907. doi: 10.1371/journal.pone.0187907 (PMC5744925; doi:10.1371/journal.pone.0187907)
Supplement: S1 File — GAPDH: glyceraldehyde-3-phosphate dehydrogenase; COL1A1: collagen, type I, alpha 1 chain; TGFB1: transforming growth factor-β1; CTGF: connective tissue growth factor; MMP2: matrix metallopeptidase 2; ACTA2: smooth muscle alpha-actin 1; PAI1A: plasminogen activator inhibitor type. (DOCX) [file pone.0187907.s001.docx]

Molecule Probe sequence Catalog number

18S TCTCAAAGATTAAGCCATGCATGTC Rn99999011_m1

COL1A1 GAGCTGCTGGCCCATCTGGTCCTAA Rn00670303_g1

TGFB1 ACCGCAACAACGCAATCTATGACAA Rn00572012_m1

CTGF CCCTGCCCTAGCTGCCTACCGACTG Rn01537278_g1

ACTA2 ACGTACAACTGGTATTGTGCTGGAC Rn01759928_G1

IL-6 AATGAGAAAAGAGTTGTGCAATGGC RN99999011_M1

IL-10 acaggtcccttgctggtggccaca RN99999012_M1

TNF ACCCTCACACTCAGATCATCTTCTC RN99999017_M1

**Box 1.** Commercial Taqman probe/primers for real time PCR (Life Technologies). GAPDH: glyceraldehyde-3-phosphate dehydrogenase; COL1A1: collagen, type I, alpha 1 chain; TGFB1: transforming growth factor-β1; CTGF: connective tissue growth factor; MMP2: matrix metallopeptidase 2; ACTA2: smooth muscle alpha-actin 1; PAI1A: plasminogen activator inhibitor type
